# Use of Tick Cell Lines in Co-Infection Studies with a Preliminary Study of Co-Culture of *Borrelia burgdorferi* and *Anaplasma phagocytophilum*

**DOI:** 10.3390/pathogens14010078

**Published:** 2025-01-15

**Authors:** Violetta Zając, Lesley Bell-Sakyi, Angelina Wójcik-Fatla

**Affiliations:** 1 Department of Health Biohazards and Parasitology, Institute of Rural Health, Jaczewskiego 2, 20-090 Lublin, Poland; afatla@poczta.onet.pl; 2 Department of Infection Biology and Microbiomes, Institute of Infection, Veterinary and Ecological Sciences, University of Liverpool, 146 Brownlow Hill, Liverpool L3 5RF, UK; l.bell-sakyi@liverpool.ac.uk

**Keywords:** *Borrelia burgdorferi*, *Anaplasma phagocytophilum*, tick cell line, co-infection

## Abstract

*Ixodes ricinus* is an important vector of infectious human and livestock diseases in Europe. Co-infections of pathogens in ticks and hosts have been reported. Tick cell lines offer a useful model system for study of co-infections. We present a review of the existing literature on co-infections in tick cell lines. Previous studies have demonstrated the usefulness of tick cell lines in studies on co-infection of different pathogens and their interaction with the tick microbiome. We also carried out a preliminary study to investigate the effects of co-culturing *Borrelia burgdorferi* and *Anaplasma phagocytophilum* on their growth and interactions with the *Ixodes ricinus* cell line IRE/CTVM19 over a 13-day period. Replication of both pathogens was quantified by real-time PCR. The presence of *A. phagocytophilum* appeared to have a slight inhibitory effect on the multiplication of *B. burgdorferi*, that were added subsequently. In contrast, the prior presence of *B. burgdorferi* appeared to have a stimulatory effect on *A. phagocytophilum* after 6 days in culture. We conclude that the IRE/CTVM19 tick cell line is suitable for simultaneous and continuous cultivation of both bacteria and can be applied in future research.

## 1. Introduction

*Ixodes ricinus*, the castor bean tick, is an important vector of a wide range of pathogens associated with human and livestock diseases. In Europe, this species is the most abundant and transmits *Borrelia burgdorferi* sensu lato, *Orthoflavivirus encephalitidis* (formerly tick-borne encephalitis virus), *Anaplasma phagocytophilum*, *Babesia* spp., and *Rickettsia* spp. [1,2]. Lyme disease (LD) in European countries is mostly caused by *Borrelia afzelii* and *Borrelia garinii* and rarely *B. burgdorferi* sensu stricto and *Borrelia bavariensis*; approximately 85,000 cases of LD are reported annually [3]. *A. phagocytophilum* is the etiological agent of human granulocytic anaplasmosis (HGA) and tick-borne fever in ruminants. In Europe, *A. phagocytophilum* is mainly transmitted by *I. ricinus*, and the seroprevalence among healthy human populations was estimated to be up to 16% [4]. It is an obligate intracellular bacterium and resides in the neutrophils of vertebrate hosts in which it causes immune suppression and increases susceptibility to secondary infections [5]. A consistent increase in the number of cases of LD and another tick-borne disease of humans, tick-borne encephalitis, has been reported, and the European Center for Disease Prevention and Control has predicted that cases of tick-borne diseases will continue to rise [6].

During feeding on an infected host, ticks can acquire pathogens that can persist throughout the molt (transstadial transmission) and, in some cases, can be transmitted from a female to her offspring (transovarial transmission). Another route of pathogen propagation in tick populations is horizontal transmission during co-feeding [7]. *I. ricinus* is known to harbor and transmit over twenty species of pathogenic or potentially pathogenic bacteria, parasites, and viruses during feeding on vertebrate hosts. The transmission of pathogens by ticks via the blood meal occurs as a result of many interactions between tick and pathogen [6]. Additionally, pathogens may help ticks to survive in unfavorable conditions [8]. Co-infections with different pathogens may occur in humans after the bite of a single tick infected by multiple pathogens and also following bites from several ticks infected by different pathogens at different times [9]. Co-occurrence of different pathogens in the *I. ricinus* species complex as well as in rodents has been confirmed in Europe, the United States, and Asia. The most frequently reported co-infections with human pathogens in these natural reservoirs are the simultaneous presence of two of *B. burgdorferi* s.l., *A. phagocytophilum*, or *Babesia* spp. [10]. Among populations of *Ixodes* spp. ticks, the rates of co-infection with *B. burgdorferi* and *A. phagocytophilum* vary from 0.5% [11] to 20.2% [12]. Lyme disease mostly occurs in temperate regions of North America, Europe, and Asia. According to the random effects model, the reported pooled global seroprevalence of *B. burgdorferi* was 14.5%, with the highest prevalences in Central Europe, Eastern Asia, and Western Europe—20.7%, 15.9%, and 13.5%, respectively [13]. Human granulocytic anaplasmosis occurs in regions with an endemic prevalence of *Ixodes* spp., and cases were described in Asia, Europe, and North America, with more serious and more frequent clinical cases reported in the United States than in Europe. The seroprevalence of *A. phagocytophilum* reported in 56 studies ranged from 0% to 37.3%, and with the use of a random-effects model, the overall pooled seroprevalence in humans was calculated as 8.4%, the highest prevalence being in high-risk populations and the lowest in healthy populations, 13.8% and 5%, respectively [4]. Simultaneous co-infection with *B. burgdorferi* and *A. phagocytophilum* has been described in humans [14,15]. Study of co-infections in engorged ticks or collected from vegetation would be difficult due to the requirement to find a large group of co-infected individuals. A substitute may be tick cell lines, which have been used in many research areas, including tick biology, physiology, immunology and genomics. These cells have found an application in the isolation and propagation of tick-transmitted viruses and bacteria and research on tick–pathogen interactions. Tick cell lines can also be used in research on anti-tick vaccines, acaricides, and in vitro study of host–vector–pathogen relationships [16,17]. Several studies have examined co-infection of different pathogenic or symbiotic microorganisms in tick cell lines including species of *Anaplasma*, *Borrelia*, *Ehrlichia*, *Rickettsia*, and *Wolbachia* [18,19,20,21,22,23,24]. The aim of this study is to investigate the effects of co-culturing *A. phagocytophilum* and *B. burgdorferi* on their growth and interactions within a tick cell line. We hypothesize that co-culturing both pathogens within the tick cell line will lead to the competitive inhibition of growth. This article additionally reviews the existing literature on use of tick cell lines in the study of co-infections.

## 2. Materials and Methods

### 2.1. Tick Cell Line

The IRE/CTVM19 cell line [16,25], derived from embryos of *Ixodes ricinus* ticks collected in England, was provided by the Tick Cell Biobank (University of Liverpool, UK). The cells were maintained in ambient air at 28 °C in L-15 (Leibovitz) medium supplemented with 20% fetal bovine serum, 10% tryptose phosphate broth, 2 mM L-glutamine, 100 units/mL penicillin, and 100 µg/mL streptomycin (all from Sigma Aldrich, St. Louis, MO, USA) with use of flat-sided culture tubes (Nunc, ThermoFisher Scientific, Waltham, MA, USA). The medium was changed once a week by removal and replacement of three-quarters of the medium volume. Subcultures were performed monthly by adding an equal volume of fresh medium to the tube, resuspending the cells by pipetting and transferring half of the suspended cells and medium into a new tube. Before subculture, the cells were observed under an inverted microscope to confirm their proper morphology and high density.

### 2.2. Infection of Tick Cell Line with B. burgdorferi and A. phagocytophilum

The IRS strain of *B. burgdorferi* Johnson et al. emend. Baranton et al. (ATCC 35211, Manassas, VA, USA) isolated from *I. ricinus* collected in Switzerland was purchased from the American Type Culture Collection (ATTC). *B. burgdorferi* was cultured in BSK-H complete medium supplemented with 6% rabbit serum (Sigma Aldrich, St. Louis, MO, USA) in a CO_2_-free incubator at 37 °C and subcultured twice-weekly at a split ratio of 1/3 by dilution in fresh medium. For inoculation of the tick cell line, one-day post-subculture bacteria (passage 32) were used.

*A. phagocytophilum* strain Harris [26,27,28], isolated from a sheep in Scotland, was donated by the Tick Cell Biobank. *A. phagocytophilum* was grown in the IRE/CTVM19 tick cell line at 28 °C in flat-sided culture tubes in medium, as described above for the tick cell line but without antibiotics. The medium was changed once a week as described for non-infected tick cells, and subculture was performed once every 3 weeks by adding 100 µL aliquots of infected cell suspension into a new tube with uninfected IRE/CTVM19 cells. The infected culture was used 1 week after subculture for inoculation at a density of 7 × 10^6^ cells per mL.

**Figure 1:**
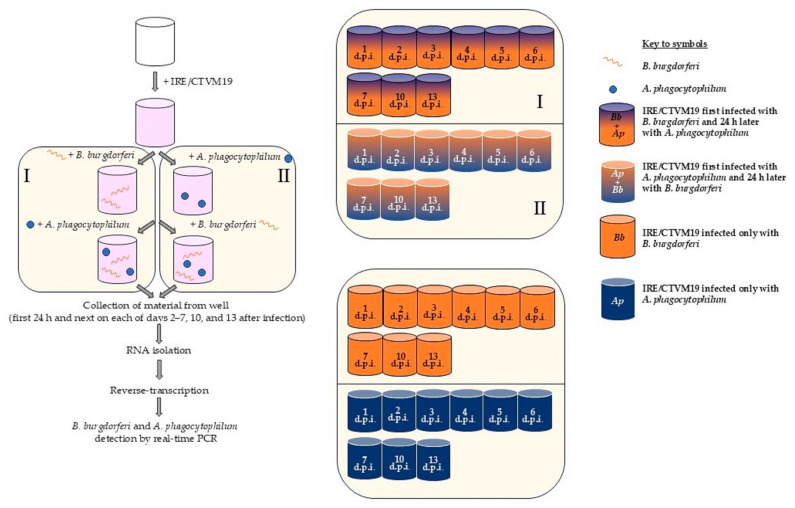
Experimental design schematic (on the **left**) and diagrams of microplates with individual wells of IRE/CTVM19 tick cell line infected by *Borrelia burgdorferi* and/or *Anaplasma phagocytophilum* and sample days after infection (on the **right**); variants of experiments: I—IRE/CTVM19 cells first infected with *B. burgdorferi* and after 24 h with *A. phagocytophilum*, II—IRE/CTVM19 cells first infected with *A. phagocytophilum* and after 24 h with *B. burgdorferi*. Abbreviations: d.p.i.—days after infection; Bb—*B. burgdorferi*, Ap—*A. phagocytophilum*.

Before the experiment, IRE/CTVM19 cells were seeded at a density of 7 × 10^5^ cells per ml in 1 mL total complete medium without antibiotics in wells of 24-well plates (Nunc, ThermoFisher Scientific, Waltham, MA, USA) and maintained overnight for attachment of cells at 28 °C. On the next day, 18 wells each were infected with 100 µL of *B. burgdorferi* suspension in BSK-H medium or with 100 µL of *A. phagocytophilum* in the IRE/CTVM19 tick cell line as described above and incubated overnight at 28 °C (Figure 1). The next day, nine of each set of infected wells were co-infected with 100 µL of the second pathogen (*A. phagocytophilum* for wells previously infected with *B. burgdorferi* and *B. burgdorferi* for wells previously infected with *A. phagocytophilum* the day before). The other nine wells of each pathogen were retained as single infection controls. The entire contents of one infected well of each treatment were harvested for RNA extraction on each of days 1–7, 10, and 13 after infection with the second pathogen (p.i.). The entire contents of the well were collected, both supernatant and monolayer. The cells were scraped from the bottom of the well using a RNAse-free sterile tip, pipetted, and collected into a sterile tube. RNA was isolated from the culture instead of DNA, because DNA analysis also detects dead bacteria, unlike RNA. On day 5 p.i., the medium in the remaining wells was changed by removal and replacement of half the volume. The uninfected culture (negative control) consisted of 3 additional wells of the plate seeded with IRE/CTVM19 cells as described above that were not infected with any bacteria. From those wells, the material was not harvested for RNA extraction but was used to compare uninfected tick cells with those infected with bacteria for all days post-infection in daily observation under an inverted microscope.

### 2.3. RNA Isolation, Reverse Transcription, and Real-Time PCR

RNA was extracted immediately after the collection of material from the well using an RNeasy Mini Kit (Qiagen, Hilden, Germany), according to the manufacturer’s protocol for animal cells. The isolated RNA was stored at −80 °C. In the next step, the total RNA was reverse-transcribed into cDNA with a QuantiTect Reverse Transcription Kit (Qiagen, Hilden, Germany). The concentration of RNA and cDNA was measured in individual samples using a spectrophotometer (NanoDrop, ThermoFisher Scientific, Waltham, MA, USA). The concentration of isolated RNA in the tested samples ranged from 107 to 151 ng/µL, and the cDNA concentration ranged from 1006 to 1572 ng/µL.

Quantification of *B. burgdorferi* and *A. phagocytophilum* in infected tick cell cultures was carried out by real-time PCR (qPCR) in samples of transcribed cDNA with Power SYBR^®^ Green PCR Master Mix (Applied Biosystems, Paisley, UK). Reactions were performed in a final volume of 25 µL, and the reaction mixture contained 12.5 µL of reaction buffer with SYBR Green, 4 µL of cDNA, 4.5 µL (*Borrelia*) or 4 µL (*Anaplasma*) nuclease-free water, and 2 µL each of primer (10 mM) for *B. burgdorferi* detection and 2.25 µL of each of primer for *A. phagocytophilum* detection. For the detection of *B. burgdorferi*, the primers FL-571F GCA GCT AAT GTT GCA AAT CTT TTC and FL-662R TGA GCT CCT TCC TGT TGA [29,30] were used, and for the detection of *A. phagocytophilum*, the primers ApMSP2f ATG GAA GGT AGT GTT GGT TAT GGT ATT and ApMSP2r TTG GTC TTG AAG CGC TCG TA [31,32] were used.

The reaction was carried out in a StepOne thermal cycler (Applied Biosystems, Paisley, UK) using the following cycle profiles: 55 °C for 2 min, 95 °C for 10 min, and 40 cycles of 95 °C for 15 s, 60 °C for 1 min, and 72 °C for 1 min for the detection of *B. burgdorferi*; 95 °C for 10 min and 40 cycles of 95 °C for 15 s and 60 °C for 1 min for the detection of *A. phagocytophilum*. Each time, a positive control was used, which consisted of genomic DNA isolated from a culture of *B. burgdorferi* or from *A. phagocytophilum* in IRE/CTVM19 cells, and a negative control in which ultrapure water was used instead of cDNA. Quantification was performed indirectly by determining the threshold cycle (Ct).

### 2.4. Statistical Analysis

The statistical significance of the differences in Ct values for individual samples depending on the day post-infection and depending on the culture variant was determined using one-way analysis of variance (One-way ANOVA). A *p* value < 0.05 was considered statistically significant.

## 3. Results

Microscopic observations of IRE/CTVM19 cultures infected with *B. burgdorferi* and/or *A. phagocytophilum* did not show any visible negative effect of bacterial infection on the tick cells compared to non-infected cultures. No tick cell lysis was observed, and cell morphology and adhesion remained unchanged throughout the 13-day experiment.

**Figure 2:**
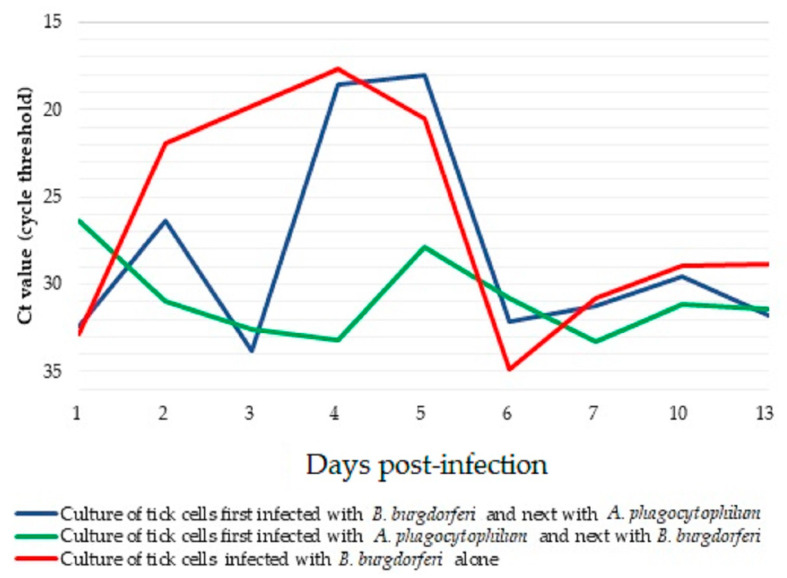
Quantification of *Borrelia burgdorferi* cDNA assessed by Ct values determined by qPCR in IRE/CTVM19 tick cell cultures to which *B. burgdorferi* was added either one day before or one day after *Anaplasma phagocytophilum*.

In the control culture of tick cells infected only with *B. burgdorferi*, the amount of spirochaete cDNA increased between days 2 and 4 p.i., while on day 5 p.i. the quantity of bacterial cDNA decreased and remained at a low level until the end of the experiment (Figure 2). In the culture infected 24 h later with *A. phagocytophilum*, the amount of *B. burgdorferi* cDNA was similar to that in the control culture, except on day 3 p.i., where the amount of bacterial cDNA decreased. However, in the culture infected first with *Anaplasma* and after 24 h with *Borrelia*, the amount of *B. burgdorferi* cDNA was higher than in the other two cultures on day 1 p.i. but was lower on subsequent days. From day 6 p.i. the amount of *B. burgdorferi* remained at a similar level in all three cultures, with no indication of further replication. The analysis did not show statistically significant differences in the Ct value between all types of cultures.

**Figure 3:**
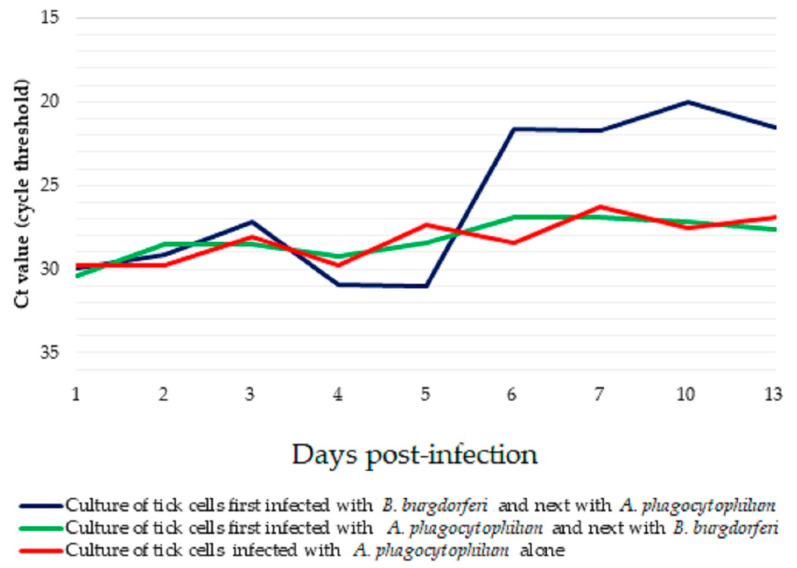
Quantification of *Anaplasma phagocytophilum* cDNA assessed by Ct values determined by qPCR in IRE/CTVM19 tick cell cultures to which *B. burgdorferi* was added either one day before or one day after *Anaplasma phagocytophilum*.

The amount of *A. phagocytophilum* cDNA was similar throughout the 13-day experiment in the control culture inoculated with *A. phagocytophilum* alone, and in the culture after subsequent infection with *B. burgdorferi*, showing only a slight, non-significant increase (Figure 3). In the culture infected first with *B. burgdorferi*, the quantity of *A. phagocytophilum* cDNA increased from day 6 p.i. and remained at a higher level than seen in the other two cultures for the rest of the experiment. Unfortunately, analysis did not show statistically significant differences in the Ct value between all types of cultures.

## 4. Discussion

Pathogens simultaneously present in one tick can affect each other and interact with tick symbionts, and these interactions can be positive when one pathogen increases the occurrence of an established microorganism or favors the emergence of an invading pathogen. Interactions can be negative when one pathogen disturbs the establishment of an infecting microorganism or promotes the extinction of an established pathogen. In most of the cases described among patients with LD and HGA, a greater number of symptoms with longer occurrence were observed compared to patients with LD alone [10]. Overall, co-infection can exacerbate the severity of disease symptoms, and difficulties in diagnosis lead to less successful treatment and more severe health outcomes [33].

Tick cell lines have been used to study co-infections with a variety of different pathogens and symbionts. Moniuszko and co-workers [20] used lines ISE6 and IRE/CTVM19 derived from *I. scapularis* and *I. ricinus*, respectively, to demonstrate the impact of one pathogen on another, depending on which pathogen (*B. burgdorferi*, *Ehrlichia ruminantium*, and Semliki Forest virus) infected the tick cells first. Tick cell lines have also found application in research on co-infection with different species from the same genus. Using the tick cell line IDE8 from *I. scapularis*, the co-infection with two *Rickettsia* species—*R. hoogstraalii* and *R. rhipicephalii*—was confirmed in *Haemaphysalis montgomeryi* ticks. The continuous replication of and competition between both species were observed [22]. Experiments conducted with the IDE8 tick cell line have shown that simultaneous infection or competition can occur between two *Anaplasma marginale* isolates. In addition, the exclusion by *A. ovis* of infection with *A. marginale* isolates was demonstrated [18].

Tick cell lines have also been used in the co-cultivation of pathogens and tick endosymbionts. Skinner and co-workers [23] demonstrated that *Wolbachia* at a higher density resulted in increased *A. phagocytophilum* proliferation and decreased immune response during culture in the ISE6 cell line. On the other hand, infection of IRE11 cells (*I. ricinus*) with the endosymbiont *Rickettsia buchneri* decreased the ability of *A. phagocytophilum*, *Rickettsia monacensis*, and *Rickettsia parkeri* to infect and replicate in the tick cell line and suggested that the tick endosymbiont has the ability to prevent transovarial transmission and colonization of *I. scapularis* by other rickettsiae [21]. Use of the ISE6 line made it possible to confirm that *Sphingomonas* spp. endosymbionts expressing the *msp*4 gene of *A. phagocytophilum* inhibited the infection of *A. phagocytophilum*. The authors suggested that this model could be applied for the prevention of *A. phagocytophilum* infection and transmission in ticks [24]. The *I. scapularis* cell lines ISE6 and IDE12 and *Dermacentor andersoni* cell line DAE15 were used to study phagocytosis of *B. burgdorferi* in the presence and absence of the tick endosymbiont *Rickettsia peacockii*, and no influence of endosymbiont was demonstrated on the phagocytosis of spirochaetes [19].

The results obtained in our preliminary *Borrelia* and *Anaplasma* co-infection experiment are consistent with the growth of *B. burgdorferi* in BSK-H medium without tick cells, which requires passage after 3–4 days of culture and was observed in other studies. Obonyo and co-workers [34] observed that *B. burgdorferi* reached the mid-logarithmic phase of growth in BSK-H medium in 4 days. We obtained similar results when *B. burgdorferi* was cultured with tick cells alone. Previous studies demonstrated the growth of *B. burgdorferi* in five tick cell lines, but the authors suggested that tick cells did not promote spirochaete growth but rather reflected differences in the media used in experiments [35]. In a later study [36], the survival and multiplication of *B. burgdorferi* were also demonstrated in primary embryonic cell cultures derived from *Rhipicephalus microplus* and *Amblyomma cajennense*. However, in the study of Moniuszko and co-workers [20], multiplication of *B. burgdorferi* with tick cells during the maximum 4-day experimental period was not detected, and differences from our results may be explained by the different strains of *B. burgdorferi* used in these two studies. In the present study, the quantity of *B. burgdorferi* decreased significantly from day 6 p.i. in all cultures, possibly due to the exchange of part of the medium. In previous studies, the spirochaetes were visualized as cell-associated and were found in the spaces between tick cells [20,34], and in our experiment, *B. burgdorferi* not associated with tick cells may have been removed during the replacement of the medium.

Our results may indicate that addition of *A. phagocytophilum* after infection with *B. burgdorferi* inhibited or delayed the onset of spirochaete replication. The inhibitory effect of *A. phagocytophilum* on *B. burgdorferi* multiplication was observed only up to the third day p.i., and the amount of spirochaete cDNA increased on the fourth day p.i., reaching a level similar to that in the control culture. Further experiments will be required to determine the reason for and extent of this inhibition. Additionally, in the case of the cultures in which *A. phagocytophilum* was the first infecting agent, we did not detect any clear increase in the amount of *B. burgdorferi* cDNA, as was observed in the control cultures and the cultures infected with *A. phagocytophilum* after infection with spirochaetes.

In general, it seems that presence of *A. phagocytophilum* may have an inhibitory effect on the multiplication of *B. burgdorferi* and that this effect is short-term in the case when it is the second infecting pathogen in vitro. In natural conditions, *A. phagocytophilum* can influence the susceptibility to secondary infection. Firstly, it causes immune suppression in the vertebrate host by its influence on immunoregulatory chemokines, proinflammatory cytokines, and/or anti-inflammatory cytokines and can result in co-infections with opportunistic microorganisms [37]. Secondly, *A. phagocytophilum* manipulates tick cell epigenetics and tick histone-modifying enzymes to regulate transcription and apoptosis in a tissue-specific manner that facilitates the development of bacteria and preservation of tick fitness [38]. Thirdly, the presence of this pathogen leads to disruption of the bacterial microfilm and reduction of the peritrophic membrane in the tick midgut by interfering with the commensal microbial community and inducing ticks to express *I. scapularis* anti-freeze glycoprotein, which may facilitate access of *A. phagocytophilum* and co-infecting pathogens to the midgut epithelium [5,39]. In tick cell cultures in vitro, *A. phagocytophilum* may use one of the last two abovementioned mechanisms to enhance the development of other pathogens. On the other hand, if it can disturb the microbial community in the tick midgut, it might also inhibit the development of other pathogens such as *B. burgdorferi*. The mechanisms described above may be responsible for the impact of *A. phagocytophilum* on the multiplication of *B. burgdorferi*. Further research with different amounts of bacteria infecting tick cells carried out with multiple replicates is necessary to confirm the influence that we observed. Additionally, future experiments on the modulation of individual elements of the tick immune response and genetic manipulations induced by *A. phagocytophilum* could explain the exact mechanisms that are involved in the effect on *B. burgdorferi*.

However, experiments on laboratory mice have shown significantly higher levels of *B. burgdorferi* presented in ear and skin samples among mice coinfected with *A. phagocytophilum* previously, compared to mice infected only with spirochaetes, whereas histopathological observation did not confirm more severe disease [40]. Another study with the use of mice simultaneously infected with both pathogens demonstrated that a higher level of spirochaetes was found in some samples of tissue in co-infected mice compared to mice infected with *B. burgdorferi* alone [41].

The usefulness of tick cell lines for the propagation of *A. phagocytophilum* was confirmed, and adhesion, internalization, and replication in tick cells were observed [42]. An increase in the *A. phagocytophilum* quantity over time after infection was observed in tick cell lines derived from *I. ricinus* and *I. scapularis* [43]. In another study, *A. phagocytophilum* strains isolated from human, canine, and ovine hosts showed one or more large vacuoles filled with numerous bacteria in the infected tick cells [44].

The usefulness of tick cell lines derived from *I. scapularis* and *I. ricinus* for propagation and study of *A. phagocytophilum* has been confirmed in multiple studies over the past three decades [23,24,28,42,43,44,45,46,47]. In our experiment, intensive multiplication of *A. phagocytophilum* was not observed, possibly because the experiment was carried out over a short period at 28 °C, and bacterial growth at this temperature is slower than at 34 °C [43]. The amount of *A. phagocytophilum* cDNA present in the tick cells was very similar in both the control cultures infected only with *A. phagocytophilum* and in cultures infected with *B. burgdorferi* after infection with *A. phagocytophilum*, which may indicate that subsequent infection with *B. burgdorferi* does not affect the in vitro growth of *Anaplasma*. The amount of *A. phagocytophilum* in these two cultures increased only slightly during the experiment. In contrast, in the culture that first received *Borrelia* and then *Anaplasma*, the amount of *A. phagocytophilum* increased from the sixth day, which could indicate stimulation of the growth of *Anaplasma* by *Borrelia* when it is the first infecting agent. Further experiments are needed to confirm this observation.

Previous studies have shown that simultaneous infection of laboratory mice with *B. burgdorferi* and *A. phagocytophilum* could enhance the pathogenesis of LD. In the case of laboratory mice infected only with *A. phagocytophilum* compared to mice subsequently infected with *B. burgdorferi*, there was no significant difference in the *A. phagocytophilum* population [40]. However, Thomas and co-workers [41] observed an increased number of neutrophils containing *Anaplasma* morulae in blood and higher levels of *Anaplasma* in tissues of co-infected mice. The same study showed that *A. phagocytophilum* had a positive effect on the transmission of *B. burgdorferi*. The authors demonstrated that humoral responses during co-infection did not affect the course of disease, whereas decreased activation of macrophages was observed [41]. The differences in the mutual influence of *A. phagocytophilum* and *B. burgdorferi* on multiplication of the second pathogen between in vitro and in vivo conditions may result from the absence of a fully-functional host immunological system during cultivation. Additionally, it was suggested that interaction between *B. burgdorferi* and *A. phagocytophilum* may be mediated by the tick and its microbiome, as both pathogens induce expression of the tick genes that facilitate infection [48]. It is impossible to fully take these factors into account in studies on tick cell lines. However, the use of tick cell lines allows the assessment of interactions with a larger amount of variance in a shorter time compared to in vivo studies. Additionally, they enable the use of genetic manipulation.

There are important limitations of the present study. Firstly, as preliminary research, the experiments were carried out only on one replicate per treatment—one well per treatment. Performing the experiment in only one repetition does not allow for the evaluation of the precision and the detection of errors. Further studies with more replicates would confirm the results obtained. Preliminary results allowed us to confirm that the methodology is effective and can be used for further research on a broader scale. Additionally, we could not determine the number of bacterial cells or MOI that were used for inoculation of the tick cells. For this reason, the results have been presented as Ct values, which enables only indirect comparison between the numbers of bacteria in different conditions and does not quantify the genome copies of the bacterium. Therefore, these results can only compare between Ct values and allow a relative quantification of bacteria depending on the day post-infection and order of infection.

In *B. burgdorferi*, at least 21 endogenous plasmids occur, and those plasmids are responsible for factors including the infectivity of bacteria. During in vitro cultivation, the loss of some plasmids was observed [49], as well as in the culture of *B. burgdorferi* in tick cell lines [50]. In this study, we did not know the plasmid content of the *B. burgdorferi* strain used, but the loss of plasmids is possible and could result in modification of the interaction with *A. phagocytophilum* compared to infective *B. burgdorferi* in vivo. Additionally, in the passage of the tick cell line before the experiment, we used a medium without antibiotics, but we could not replace all of the medium. The residual penicillin could have influenced *B. burgdorferi* growth, as penicillin shows quite potent activity against the stationary-phase and growing forms of spirochaetes [51]. The final limitation was the use in experiments of the tick cell line, which does not fully reproduce conditions in an intact tick. The differences that could influence the co-culture of bacteria in vitro are the absence of humoral and cellular responses and the tick microbiome when using only tick cells. These factors are involved in developing infection in vivo, and the use of cell lines does not allow for full reconstruction of the interactions that occur in natural conditions.

## 5. Conclusions

The results obtained in our preliminary co-infection study indicate that *A. phagocytophilum* might inhibit multiplication of *B. burgdorferi*, whereas *B. burgdorferi* could enhance the level of *A. phagocytophilum* during in vitro culture with tick cells. However, the mutual influence on growth of the second pathogen in tick cell lines requires further studies in vitro to confirm these interactions, as well as in vivo where humoral and cellular responses and the microbiome of ticks can be taken into account. Future research in vitro using the tick cell line with different amounts and proportions of co-infecting bacteria could confirm our results. It will be essential to conduct further research using several replicates within each experiment, repeating the experiments and evaluating the quantity of bacteria in each day post-infection with the incorporation of absolute methods. This would allow for more precise results with a lower risk of error. Subsequent stages of the co-infection study could also include in vivo experiments using laboratory ticks simultaneously infected with *B. burgdorferi* and *A. phagocytophilum* by acquisition feeding on artificial membranes and transmission feeding on laboratory animals. The use of whole ticks to study co-infections would allow for the inclusion of immunological mechanisms in the assessment of the mutual influence of bacteria. Such future experiments, carried out under in vitro and in vivo conditions and using molecular methods, could answer the question of what specific mechanisms are involved in interactions between co-infecting bacteria, both in the vector and host organisms.

We conclude that the tick cell line IRE/CTVM19 derived from *I. ricinus* is suitable for simultaneous and continuous cultivation of *B. burgdorferi* and *A. phagocytophilum*, and this cell line could be used for future studies of pathogen–pathogen and pathogen–vector interactions. Previous reports have proved the usefulness of this and other tick cell lines in studies on the co-infection of pathogens and symbionts and application of this in vitro model for future research.

## Data Availability

The data presented in this research are available in the manuscript.
